# Non-thermal phonon dynamics and a quenched exciton condensate probed by surface-sensitive electron diffraction

**DOI:** 10.1038/s41563-024-01880-6

**Published:** 2024-04-30

**Authors:** Felix Kurtz, Tim N. Dauwe, Sergey V. Yalunin, Gero Storeck, Jan Gerrit Horstmann, Hannes Böckmann, Claus Ropers

**Affiliations:** 1 Department of Ultrafast Dynamics, Max Planck Institute for Multidisciplinary Sciences, Göttingen, Germany; 2 4th Physical Institute, Solids and Nanostructures, University of Göttingen, Göttingen, Germany; 3 Department of Materials, ETH Zurich, Zurich, Switzerland

**Keywords:** Structure of solids and liquids, Surfaces, interfaces and thin films, Imaging techniques, Phase transitions and critical phenomena

## Abstract

Interactions among and between electrons and phonons steer the energy flow in photo-excited materials and govern the emergence of correlated phases. The strength of electron–phonon interactions, decay channels of strongly coupled modes and the evolution of three-dimensional order are revealed by electron or X-ray pulses tracking non-equilibrium structural dynamics. Despite such capabilities, the growing relevance of inherently anisotropic two-dimensional materials and functional heterostructures still calls for techniques with monolayer sensitivity and, specifically, access to out-of-plane phonon polarizations. Here, we resolve non-equilibrium phonon dynamics and quantify the excitonic contribution to the structural order parameter in 1T-TiSe_2_. To this end, we introduce ultrafast low-energy electron diffuse scattering and trace strongly momentum- and fluence-dependent phonon populations. Mediated by phonon–phonon scattering, a few-picosecond build-up near the zone boundary precedes a far slower generation of zone-centre acoustic modes. These weakly coupled phonons are shown to substantially delay overall equilibration in layered materials. Moreover, we record the surface structural response to a quench of the material’s widely investigated exciton condensate, identifying an approximate 30:70 ratio of excitonic versus Peierls contributions to the total lattice distortion in the charge density wave phase. The surface-sensitive approach complements the ultrafast structural toolbox and may further elucidate the impact of phonon scattering in numerous other phenomena within two-dimensional materials, such as the formation of interlayer excitons in twisted bilayers.

## Abstract (web summary)

The thermalization of acoustic phonons after photoexcitation is traced by electron pulses in TiSe_2_, and the excitonic contribution to the structural order parameter of the material’s charge density wave phase is quantified.

## Main

Two-dimensional (2D) materials, such as graphene and transition metal dichalcogenide monolayers, promise unique functionality via enhanced correlations and tunability in stacked heterostructures^1–3^. Many features of such systems result directly from the highly anisotropic nature of the layered parent materials, which is evident in their electrical and thermal properties. Specifically, weak interlayer couplings cause a quadratic dispersion of out-of-plane polarized acoustic phonons^4^. The resulting enhanced low-frequency phonon density of states negatively impacts the electronic mobility, unless symmetries suppress carrier–phonon scattering^5^. Notably, anharmonicity of these modes causes the unusual in-plane thermal contraction of graphene^6^. Moreover, the effectively reduced dimensionality in layered compounds also favours the formation of charge density waves (CDWs) and other correlated phases via strong couplings between electronic and lattice degrees of freedom. Whereas most of the mechanical properties and the dispersion of lattice modes are universal features of layered structures, such correlated behaviour is highly material dependent^7^. For instance, titanium diselenide (1T-TiSe_2_), a prominent member of the transition metal dichalcogenide family, shows the peculiar phenomenon of exciton condensation^8,9^ conspiring with a Peierls mechanism^10,11^ to form its CDW state^12,13^.

Elucidating the origins of emergent states, as well as quantifying the coupling of elementary excitations, is rather challenging in thermal equilibrium. To reveal microscopic interactions, ultrafast measurement techniques create a non-equilibrium situation by photoexcitation, which is subsequently tracked on the intrinsic femtosecond to picosecond timescales of electronic and lattice thermalization. Probes with complementary sensitivities yield a comprehensive understanding, and, in particular, lattice dynamics are elucidated by ultrafast diffraction based on electron or X-ray pulses^14–25^. The intensity and width of diffraction peaks are measures of long-range structural order, whereas the inelastic scattering background can reveal transient phonon populations, as recently explored in ultrafast electron diffuse scattering^26–31^ (UEDS). To date, time-resolved diffuse electron scattering studies have mainly concentrated on thin-film transmission at keV to MeV energies with limited access to the unique features of 2D materials. The here presented approach addresses this issue by employing low-energy electron pulses with high surface sensitivity in a reflection geometry.

In this Article, we target two essential aspects of non-equilibrium structural relaxation in TiSe_2_: Introducing ultrafast low-energy electron diffuse scattering (ULEEDS), we reveal non-thermal lattice dynamics by tracking out-of-plane polarized acoustic (ZA) phonons. Being elusive in transmission diffraction, we expect a broad application of this approach to other layered materials. Moreover, beyond the mapping of fluctuation modes, we address the intricate nature of the CDW phase in TiSe_2_ by analysing a transient quench of the superstructure, thus disentangling excitonic effects from the Peierls instability.

## Ultrafast low-energy electron diffraction

**Fig. 1:**
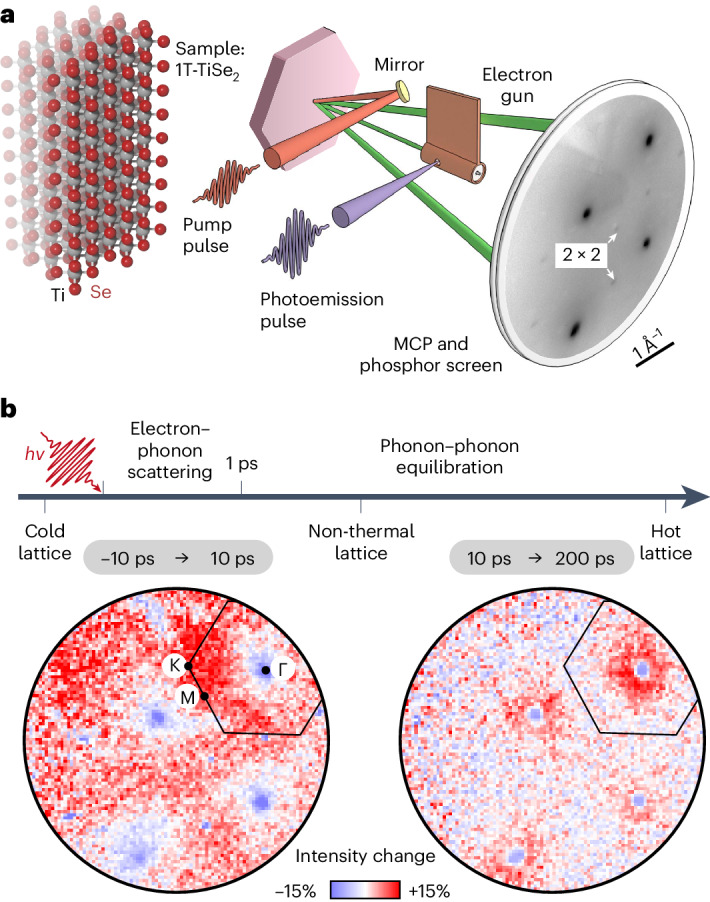
The ULEED setup and transient dynamics within diffraction images of TiSe_2_. **a**, The experimental scheme, crystal structure and a diffraction image of 1T-TiSe_2_ at 30 K (electron energy 86 eV). Main lattice peaks are clearly visible, and superstructure spots are highlighted by arrows. MCP, microchannel plate assembly. **b**, Fractional intensity changes between three different pump–probe delays, displayed as *I*(*k*_*x*_, *k*_*y*_, Δ*t*_2_)/*I*(*k*_*x*_, *k*_*y*_, Δ*t*_1_) − 1: First, the intensity of the main lattice peaks (and superstructure reflexes) is reduced, while the background increases primarily at the Brillouin zone boundary (black). At larger delays, a further suppression of the main lattice peaks is accompanied by an increase only in their vicinity. After 200 ps, only minor changes are observed before cooling sets in on a nanosecond timescale.

The experiments were conducted using a recently developed ultrafast low-energy electron diffraction (ULEED) apparatus, which allows for tracing structural dynamics at surfaces, for example, CDW phase transitions^32–35^. In this approach, low-energy electron pulses are generated from a nanoscopic needle emitter via two-photon photoemission and collimated in a miniaturized electrostatic lens geometry^36^. The electron pulses are backscattered from the investigated sample surface, allowing for a laser pump/electron probe scheme (Fig. 1a) with temporal resolution down to 1 ps at electron energies of about 20–200 eV. The strongly confined emission area of the tip emitter leads to a high transversal coherence length of the electron probe, resulting in a momentum resolution below Δ*k*_s_ = 0.07 Å^−1^. Using a phosphor screen microchannel plate assembly and a complementary metal oxide semiconductor camera, backscattering electron images *I*(*k*_*x*_, *k*_*y*_, Δ*t*) are recorded as function of the pump–probe delay Δ*t*. Here, the coordinates (*k*_*x*_, *k*_*y*_) denote detector positions relating to the transverse momentum change upon scattering.

In the present work, we study the surface of a TiSe_2_ single crystal consisting of van der Waals-bonded Se–Ti–Se trilayers (Fig. 1a). Upon cooling the sample to 30 K, well below the critical temperature *T*_c_ = 200 K, the material undergoes a second-order phase transition to the (2 × 2 × 2)-reconstructed commensurate CDW state^37,38^ (apparent in the low-energy electron diffraction pattern in Fig. 1a), accompanied by a band gap opening of about 100 meV (ref. ^7^). Furthermore, the initial phonon population is substantially reduced. Femtosecond, near-infrared excitation of the electronic system, in turn, reverses the phase change, preferentially populates the in-plane polarized amplitude mode and creates a broad non-thermal optical phonon distribution^39–42^. We track the ensuing lattice dynamics with monolayer surface sensitivity by harnessing the large scattering cross section at low electron energies^43^ and the resulting high signal-to-noise ratio. In particular, we follow the evolution of the diffuse scattering background (‘Phonon dynamics’ section) and the CDW superstructure reflexes (‘Charge density wave dynamics’ section).

## Phonon dynamics

In ultrafast diffraction, transient phonon populations can be revealed in momentum-resolved maps of the inelastic scattering background. Such maps enable the extraction of the strength of electron–phonon and phonon–phonon couplings across the Brillouin zone, as demonstrated by the recently developed UEDS technique^26–29^. We transfer this concept to backscattering diffraction (ULEEDS) and discuss its implications regarding the sensitivity to specific phonon branches.

A first insight into the optically induced lattice dynamics can be obtained by comparing the intensity distributions from three selected pump–probe delays. Shortly after photoexcitation (Δ*t* = 10 ps; Fig. 1b, left), the main lattice peaks are partially suppressed. Simultaneously, we find a redistribution of diffraction intensity to regions farther from the peaks. Between delays of 10 and 200 ps (Fig. 1b, right), a further reduction of the spot intensity occurs, but now accompanied with a pronounced intensity increase only in close vicinity to the main lattice peaks.

**Fig. 2:**
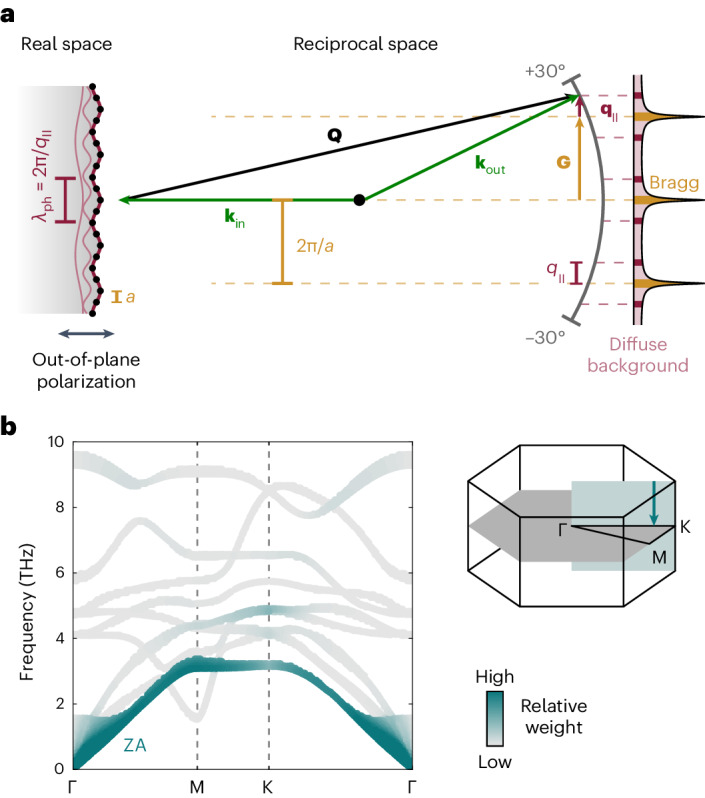
Sensitivity of ULEEDS. **a**, The scattering geometry and a real-space depiction of a phonon contributing to the diffuse background. Incident, outgoing and scattering vectors are denoted by **k**_in_, **k**_out_ and **Q**, respectively. **b**, Phonon dispersion of TiSe_2_ and the colour-coded relative contribution of each phonon mode to the diffuse background, obtained for a thermal state. Here, we normalize the contributions across all nine phonon branches and the out-of-plane momentum, as illustrated in a sketch of the Brillouin zone (right). The experiment is particularly sensitive to the out-of-plane polarized acoustic branch (ZA).

The measured background signal arises from the inelastic scattering of incident electrons by specific phonons, as illustrated schematically in Fig. 2a. In a first approximation (one-phonon scattering), the scattering vector **Q** = **k**_out_ − **k**_in_ is directly linked to the phonon momentum **q**_∣∣_, such that the structure of the background reveals the phonon population^26–29^. Therefore, our observations in Fig. 1b evidence a transient non-thermal state of the lattice, which is characterized by slower phonon creation at the centre of the Brillouin zone as compared with the zone boundary.

In a further analysis, we determine which phonon modes contribute to the diffuse background. In the mentioned limit of one-phonon scattering^18^, the diffuse intensity *I*_1_(**Q**, Δ*t*) is proportional to ${\sum }_{\nu }\frac{1}{{\omega }_{{{{\bf{q}}}}\nu }}(2{n}_{{{{\bf{q}}}}\nu }+1){\left\vert {F}_{\nu }({{{\bf{Q}}}})\right\vert }^{2}$, with *n*_**q***ν*_, *ω*_**q***ν*_ and *F*_*ν*_(**Q**) being the time-dependent population, frequency and structure factor of a phonon mode belonging to branch *ν*, respectively. It becomes apparent that the largest contribution to the diffuse scattering stems from low-frequency modes. The structure factor contains the scalar product of the scattering vector and the atomic displacements associated with the particular phonon mode (for further details, see Methods), such that the scattering geometry determines the accessible phonon branches. In transmission experiments, diffraction angles are typically small and there is little out-of-plane momentum transfer, yielding a higher sensitivity to in-plane polarized phonons. In contrast, in a backscattering geometry, the scattering vector is nearly twice the incident free-electron wave vector **k**_in_ in length, pointing out of the surface. In our experimental setup, we collect the scattered electrons within an angle of *α* = ±30° to the surface normal, yielding a maximum reduction in the out-of-plane component of the scattering vector by only $1-\cos (\alpha /2)=4\, \%$. This preserves its out-of-plane character throughout the diffraction image and explains why we do not observe notable differences between the individual recorded Brillouin zones. Notably, the geometry features a particularly large out-of-plane momentum transfer of approximately 9.5 Å^−1^ at an electron energy of 86 eV. Using phonon properties retrieved from ab initio density functional theory (DFT) calculations and scattering amplitudes of the individual atoms, we are able to calculate the relative contribution of each phonon mode to the diffuse background (Methods). The calculations show that the ZA branch will dominate the signal (Fig. 2b).

**Fig. 3:**
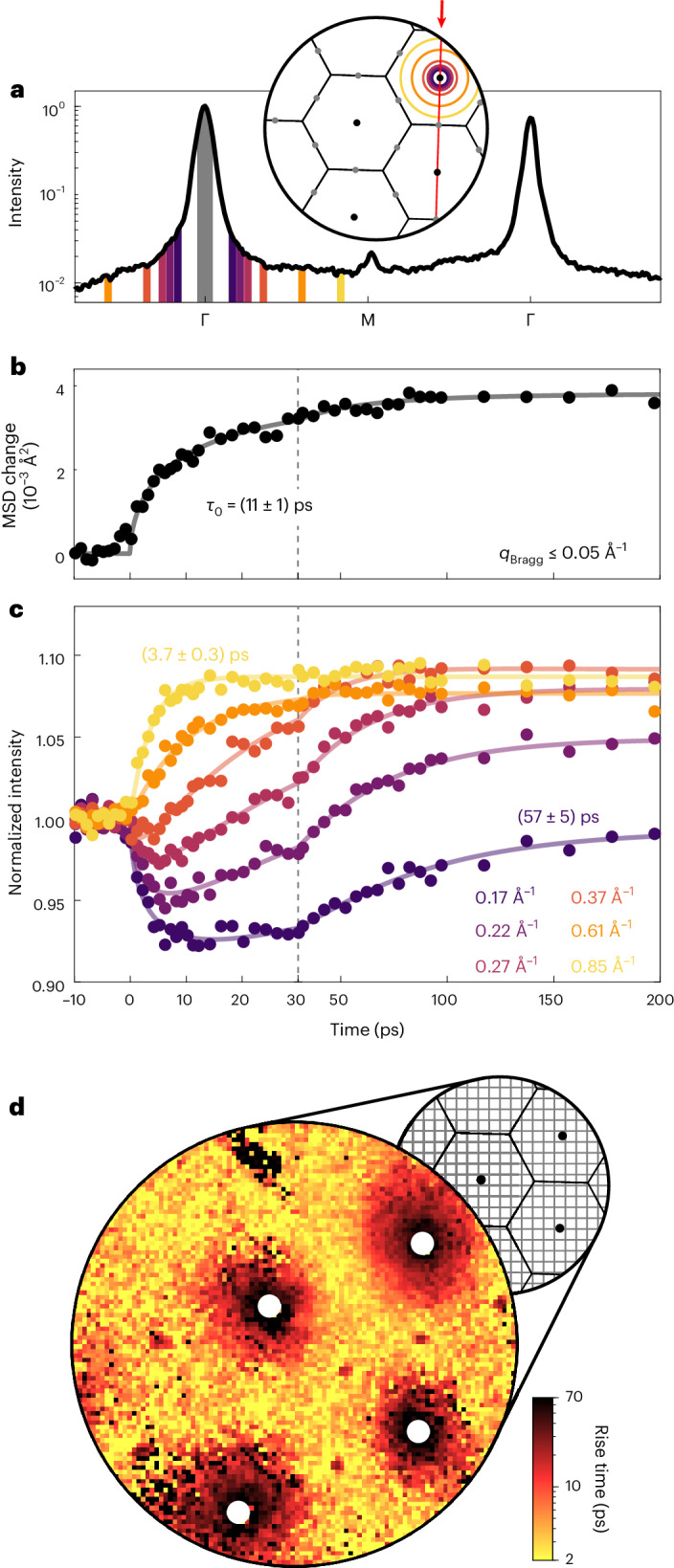
Transient momentum-resolved intensity redistribution due to an increased phonon population. **a**, Intensity profile intersecting two main lattice spots (as indicated in the schematic diffraction pattern). **b**, Temporal evolution of the change in MSD deduced from the suppression of the main lattice spots, fitted by a stretched exponential function. **c**, Temporal evolution of the intensity integrated in rings around the main lattice spot, as indicated in **a**. The time constants for the phonon build-up are extracted by a fit with equation (7), showcasing a strong momentum dependence. **d**, A map of phonon rise times extracted in a pixelwise analysis (where one pixel corresponds to 0.05 Å^−1^). Rise times at the main lattice spot locations cannot be determined (white circular areas).

**Fig. 4:**
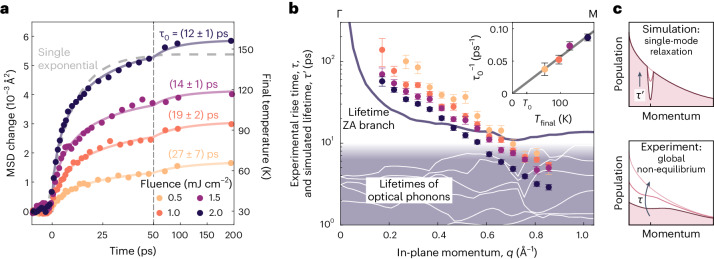
Accelerated phonon thermalization for increased excitation. **a**, Evolution of the MSD change for four different fluences. Fits with stretched exponentials (equation (6)), sharing a common stretching exponent, return the stated time constants *τ*_0_ with 1*σ* confidence intervals (dashed grey line showing a single exponential fit). Right axis: the temperature assignment according to the measured MSD change (for the performed calibration, see Methods). **b**, Momentum-resolved time constants *τ*_*q*_ of the phonon build-up for the same four fluences, compared with phonon lifetimes ${\tau }_{q}^{{\prime} }$ limited by phonon–phonon scattering (150 K). The error bars indicate 1*σ* confidence intervals of the fit. For the lowest fluence, the two values at the smallest momenta are omitted owing to their large uncertainties. Inset: the rate of MSD increase ${\tau }_{0}^{-1}$ showcases a proportionality with the final temperature *T*_final_, where *T*_0_ marks the base temperature of 30 K. The error bars indicate 1*σ* confidence intervals of the fit shown in **a**. **c**, A sketch illustrating the simplified relaxation time approximation employed in the simulations (top) versus the complete population dynamics ensuing in the experimental scenario (bottom), which entails cascaded scattering events.

In the following, we quantitatively analyse the two most prominent features within the delay-dependent series of diffraction images, namely (1) the suppression of the main lattice peaks and (2) the accompanying increase of the diffuse background intensity leading to momentum-resolved rise times of the phonon population. The dynamics of (1) give us a measure of the increase in the out-of-plane mean-squared displacement (MSD) $\langle {u}_{\perp }^{2}\rangle$ via the Debye–Waller effect^43^ ${I}_{{{{\rm{main}}}}}(\Delta t)={I}_{{{{\rm{main}}}},0}\times \exp \left(-{Q}^{2}\times \Delta \langle {u}_{\perp }^{2}\rangle (\Delta t)\right)$. As such, it represents an average over all phonon momenta and branches exhibiting substantial out-of-plane polarization. Figure 3b shows the transient increase in the MSD, which quickly rises within the first 5 ps but stretches out over more than 30 ps. This dynamic is noticeably non-exponential (see also Fig. 4a). Instead, it is well described by a stretched exponential function, requiring only one additional fit parameter. Interpreting this function as a superposition of simple exponentials^44^ suggests that the phonons contributing to the MSD differ substantially in their rise times. Hence, we expect a multitude of timescales observable within the diffuse background.

Previous studies at room temperature and in transmission reported an increase of the in-plane MSD, which occurs largely within the first picoseconds^30,45^. This rapid energy transfer from the electronic system to the lattice is also preserved in the CDW phase, as time- and angle-resolved photoemission spectroscopy and reflectivity measurements observe a similarly fast relaxation of the electronic temperature^40–42^. However, such methods are less sensitive to the here observed ensuing thermalization within the lattice. Comparing our findings with the reported timescales, we conclude that the ZA phonons are only weakly coupled to the electronic system and are consequently created predominantly via phonon–phonon scattering.

Hereafter, we deduce momentum-dependent rise times of the phonon population by analysing the transient intensity increase of the diffuse background. As the redistributed intensity from the main lattice spot is spread across the entire Brillouin zone, the absolute intensity change therein is small and some averaging is necessary to enhance the signal. We thus utilize the apparent radial symmetry around the diffraction spots by integrating the intensity within rings (sketched in Fig. 3a), effectively summing up all phonons with the same wavenumber *q* = ∥**q**_∣∣_∥. For simplicity, the analysis is restricted to the top right Brillouin zone, which features the strongest scattering signal. In the vicinity of the main lattice spot, it is important to distinguish between elastically and inelastically scattered electrons. In particular, the diffuse background is almost two orders of magnitude weaker than the peak (see the intensity profile in Fig. 3a), and tails of the latter could superimpose the inelastic intensity recorded in its surrounding. Here, we disentangle this contribution based on its temporal behaviour. The intensity evolution within selected rings is shown in Fig. 3c, exhibiting dynamics over a wide range of timescales. Whereas the intensity close to the zone boundary rises only within the first picoseconds, the evolution in the three innermost rings is more complex: Initially, the intensity decreases rapidly, followed by a slow increase. We attribute the former to the suppression of the Bragg contribution and the latter to the build-up of phonons, which converge towards an elevated thermalized population. Moreover, a third, time-independent contribution has to be considered, stemming from the already present phonon population before photoexcitation, scattering at static disorder of the sample as well as higher energy losses. Accounting for these contributions (Methods), we extract momentum-resolved rise times *τ*_*q*_ of the phonon population, which differ for the innermost and outermost of the selected rings by more than one order of magnitude.

The performed analysis of the radial intensity distribution is complemented by a pixelwise analysis of the diffraction images in a fine two-dimensional momentum grid. Note that this approach does not require any knowledge on symmetries within the diffraction pattern. A map of the extracted rise times (Fig. 3d) exhibits similar behaviour around all four main lattice spots, evincing radial symmetry, which validates the ring-wise integration applied for future analysis.

Further evidence for phonon–phonon coupling as the dominant physical mechanism observed is obtained from fluence-dependent measurements. The evolution of the MSD is shown in Fig. 4a. An increased fluence not only leads to an increased final temperature of the lattice but also accelerates the thermalization, indicated by a decreasing *τ*_0_. This is explained by increased phonon–phonon scattering rates as their population grows^46^, whereas electron–phonon scattering does not exhibit a strong dependence on excitation strength^47^. The phonon build-up rate 1/*τ*_0_ is to first order proportional to temperature^46^, in good agreement with our measurements (Fig. 4b, inset). This acceleration is observed throughout the entire Brillouin zone, as visualized by the obtained momentum-resolved rise times *τ*_*q*_ (Fig. 4b). We identify a nearly exponential decrease of the measured rise time with momentum, which uniformly drops with the absorbed fluence. Overall, these rise times are consistent with the corresponding decay time *τ*_0_ of the main lattice spot, which represents an average rise time (values stated in Fig. 4a).

To contrast the measured rise times *τ*_*q*_ with theoretical expectations on the timescale of phonon–phonon interactions, we computed phonon lifetimes ${\tau }_{q}^{{\prime} }$ via density functional perturbation theory (Methods). In this approach, the calculation predicts relaxation times for a single mode initially populated higher or lower than that of an otherwise equilibrated phonon system at a given temperature (a relaxation time approximation; Fig. 4c, top sketch). This represents a simplification compared with the experimental situation, where practically all modes exhibit a transiently evolving non-equilibrium population (Fig. 4c, bottom sketch). The solid lines in Fig. 4b display the computed lifetimes for the ZA acoustic branch (dark line) and the optical phonon branches (white lines in the shaded area), using a bath temperature of 150 K, which corresponds to the final temperature in the experiment with the highest fluence (darkest symbols; see also Fig. 4a, right axis). For the ZA branch, we find diverging lifetimes at the zone centre, a sharp drop with increasing momentum and a nearly constant value near the zone boundary. In contrast, the lifetimes of the optical phonon branches are not monotonic in momentum and mostly lie well below 10 ps, which implies a rapid equilibration among these phonons. The range of predicted lifetimes is in overall agreement with the experimentally measured rise times, and both experiment and simulation exhibit longer timescales towards the zone centre.

However, there are notable deviations in these quantities that directly arise from the non-equilibrium character of the experiment (Fig. 4c, bottom). Specifically, at large momenta, the fast and fluence-dependent rise times may indicate that a subset of modes is initially populated to a high degree. In addition, some sensitivity to the short-lived optical phonons should also be considered (cf. Fig. 2b). Near the zone centre, we find rise times slower than expected from the relaxation time approximation, which might be understood based on sequential scattering events. In particular, phonon–phonon scattering processes creating a population near the zone centre often involve low-energy phonons that have small momenta themselves. This suggests cascaded processes to fully thermalize the population near the Γ point, substantially slowing down the observable rise times, specifically when compared with relaxation times simulated for a thermal system. Relating these observations to more detailed theoretical predictions may be accomplished by using an explicit non-equilibrium theoretical approach, such as the Boltzmann transport equation^48–50^, which is, however, beyond the scope of the present work.

## Charge density wave dynamics

**Fig. 5:**
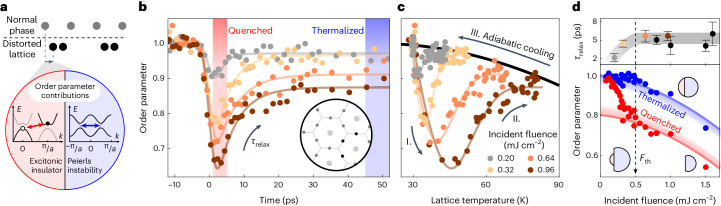
Transient dynamics of the charge density wave. **a**, In TiSe_2_, an exciton condensate and a Peierls instability contribute to the PLD. **b**, Evolution of the structural order parameter for four different fluences, as stated in **c**. The rapid initial quench is followed by a relaxation to a quasi-thermalized value at an elevated temperature. The schematic diffraction pattern illustrates the positions of the superstructure reflexes (dark grey) and the most intense ones analysed (black). **c**, Structural CDW order parameter versus lattice temperature deduced from the transient Debye–Waller factor, illustrating the non-thermal separation of the CDW quench and the increasing phonon population. **d**, Fluence dependence of the relaxation time (top) as well as the order parameter value in the quenched and quasi-thermalized stage (bottom). At a fluence of *F*_th_ = 0.5 mJ cm^−^^2^, the quenched PLD amplitude exhibits a distinct kink, and the relaxation time *τ*_relax_ is largely independent of the fluence beyond this threshold. Circle diagrams illustrate the quenched and relaxed amplitudes of both contributions to the order parameter. The smaller, excitonic component is particularly soft; that is, during the quench it is already fully suppressed at *F*_th_. Error bars in the top panel indicate 1*σ* confidence intervals of the fitted relaxation time.

Beyond the investigation of lattice thermalization, we further address the laser-induced quench of the charge-ordered phase. Notably, nearly energetically aligned electron and hole pockets at the L and Γ points of the Brillouin zone render TiSe_2_ unstable against periodic distortions with the corresponding difference wave vector. These include both a lattice modulation driven by electron–phonon coupling^10,11^ and a charge modulation driven by excitonic correlations^8,9^, which in turn will affect the lattice distortion. Jointly occurring in thermal equilibrium (Fig. 5a), time-resolved studies may disentangle these two contributions^13,39–41,51–55^. In our experiment, the structural order parameter of the phase transition is encoded in the intensity of the corresponding superstructure peaks (accounting also for the Debye–Waller factor; Methods). We are particularly concerned with the following two questions: Does the fluence-dependent speed-up of lattice thermalization affect the quench and relaxation of the order parameter? How does the exciton condensate manifest in the structural distortion at the surface, respectively, in a single layer?

We find that the structural order is transiently quenched and largely recovers within about 20 ps to a quasi-thermalized, partially suppressed value (Fig. 5b). Occurring on a similar timescale as the thermalization of the overall lattice, we analyse a possible correlation of CDW and phonon dynamics in fluence-dependent measurements. By plotting the evolution of the order parameter versus the momentary lattice temperature (Fig. 5c), we trace the non-thermal dynamics before the return to the equilibrium line (black) and an adiabatic cooling on the nanosecond timescale sets in. For low fluences, the order parameter recovers nearly completely before lattice thermalization takes place, such that these processes are decoupled in time. At higher fluences, one may expect that the faster lattice equilibration will also accelerate the recovery of the order parameter, for example, via stronger damping of the amplitude mode or faster electronic relaxation by stimulated phonon scattering. However, we find that the CDW recovery time is remarkably fluence independent beyond a threshold *F*_th_ = 0.5 mJ cm^−^^2^, at around 5 ps, being somewhat faster only for the lowest fluences (Fig. 5d, top).

It is instructive to more closely consider the fluence dependence of the order parameter in both the quenched and the partially recovered states (Fig. 5d, bottom). Whereas an effectively thermal behaviour is found beyond 50 ps (blue line), the initial quench exhibits a distinct kink at *F*_th_, such that, at higher fluences, a further suppression of the lattice distortion becomes less efficient in terms of excitation density. Both findings imply that an electronic contribution to the order parameter is fully suppressed beyond *F*_th_. It is apparent that this component is indeed the structural manifestation of the excitonic order, which, based on previous terahertz spectroscopy experiments, can be completely quenched while some periodic lattice distortion (PLD) remains^13^. Structural implications of this quenched exciton condensate were recently reported based on transmission diffraction experiments and discussed in terms of a break-up of interlayer correlations^55^. The associated spreading of diffraction intensity along the M–L line renders a quantitative determination of the PLD amplitude challenging in bulk transmission, unless tilt-series tomographic diffraction is carried out^25^. The particular surface sensitivity of ULEED, however, almost exclusively probes the PLD in a single trilayer, physically averaging over the entire L–M–L edge of the Brillouin zone. Beyond a loss of interlayer correlations, our measurements thus show that the complete suppression of excitonic order also causes a substantial weakening of the PLD amplitude within each layer. We estimate that the exciton condensate contributes about 30% to the total structural order parameter, that is, the amplitude of the lattice distortion (Methods). Our interpretation is further supported by converting the value of *F*_th_ to the absorbed energy density to allow for a comparison with literature values (note that the stated fluences are incident fluences). On the basis of the measured temperature increase and the material’s specific heat^56^, we calculate a deposited energy of 5.5 meV per normal-state unit cell. This value generally agrees with the critical fluences encountered in several time-resolved studies^13,39,41,53–55^, whereby some deviation must be expected owing to differing base temperatures, surface- versus bulk-sensitive techniques and a general uncertainty in fluence estimation.

With respect to the 30:70 split of the order parameter into excitonic and Peierls contributions, the nearly constant recovery time beyond *F*_th_ appears to represent an intrinsic formation time of the exciton condensate, while a faster recovery follows from a weaker perturbation of the condensate. A related observation was made in terahertz spectroscopy^13^, with a reported faster relaxation time of 1.5 ps. This indicates a minor temporal lag of the structural distortion in response to the electronic signature, which may result from a re-establishment of interlayer correlations^25,55^.

## Conclusions

In this work, we used the ultimate surface sensitivity of low-energy electron diffraction to investigate non-thermal phonon dynamics and the structural component of an excitonic quench in a correlated 2D material. Specifically, we introduce ULEEDS to attain a momentum-resolved view on the cascaded build-up of ZA phonons. The revealed wide range of time constants, ranging from a few picoseconds up to 100 ps towards the zone centre, manifests itself also in the non-exponential increase of the mean squared atomic displacement. Since this quantity is easily accessible in all ultrafast diffraction experiments, it can serve as a simple indicator for scenarios where a more in-depth investigation of diffuse scattering is warranted. Moreover, the accelerated equilibration with increased excitation density allows us to identify the thermalization mechanism of the ZA phonons, namely phonon–phonon interactions rather than a direct coupling to the electronic system. Related equilibration processes are expected for most layered materials. In comparison, the structural fingerprint of the excitonic order in TiSe_2_ is rather unique. It is found to be particularly susceptible to photodoping and contributes approximately 30% to the total lattice distortion, which is otherwise governed by a Peierls mechanism. Both findings highlight the importance of a combined approach involving methods sensitive to lattice as well as electronic degrees of freedom, allowing a comprehensive picture of thermalization in anisotropic materials. Given its surface sensitivity, ultrafast low-energy electron scattering represents a suitable probe to study the dynamics of surface-bound phonons^57^, chiral phonons^58–60^ and structural non-equilibrium associated with phonon-mediated charge transfer in heterostructures and moiré superlattices^24^.

## Methods

### ULEED measurements

**Extended Data Fig. 1:**
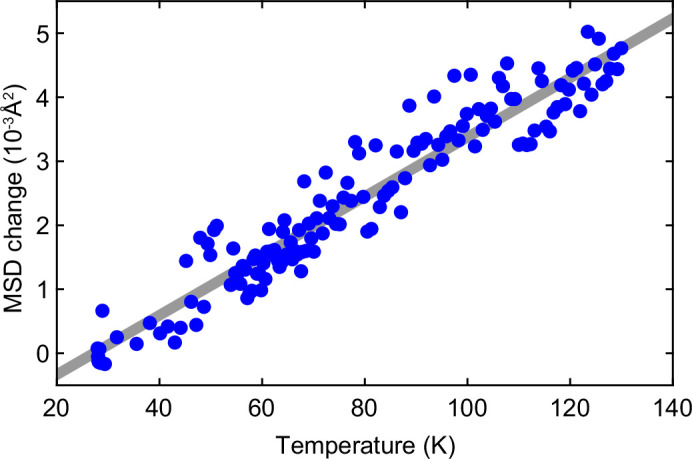
Temperature calibration necessary for time-resolved measurements. Adiabatic heating of the sample while recording the strength of the main lattice peaks yields the relationship between MSD change and temperature (blue dots, data; grey line, linear approximation used thereafter as the calibration of the pump-probe measurements).

The experiments are conducted using a recently developed ULEED apparatus^36^ that allows for tracing structural dynamics at surfaces, including charge density wave phase transitions^32–35^ and energy transfer at molecular overlayers^61,62^. In all the presented experiments, the probing electrons have an energy of 86 eV and were generated by 400 nm laser pulses (40 fs duration) via two-photon photoemission. The electron probe beam has a diameter on the sample of 10–20 μm. The TiSe_2_ sample (purchased from HQ Graphene, diameter 3–4 mm, thickness >300 μm) is cleaved under ultra-high vacuum conditions (base pressure of 2 × 10^−10^ mbar) and cooled to a temperature of 30 K using a liquid helium cryostat. Photoexcitation is provided by 200 fs laser pulses at a repetition rate of 100 kHz (central wavelength 1,030 nm, spot diameter 200 μm). Considering the larger lateral size of the pump laser and its deeper penetration depth^63^ of around 20 nm, in contrast to 5–10 Å for the low-energetic electrons^43^, we ensure probing of a homogeneously pumped sample area. The repetition rate exceeds that of most ultrafast electron diffraction experiments, which is possible owing to a good thermal contact to the underlying bulk material. This allows for a high signal-to-noise ratio and tolerable integration times of the diffraction patterns of 1–3 min per pump–probe delay. Temperature calibration of the pump–probe measurements (Fig. 4) is performed by adiabatic heating of the sample while continuously taking diffraction images. The decreasing intensity of the main lattice spots yield the temperature dependence of the increasing MSD (linear approximation, shown in Extended Data Fig. 1).

### Diffuse scattering theory

Ultrafast diffraction based on electron or X-ray pulses^64,65^ is a powerful method for studying non-equilibrium lattice dynamics. The intensity and width of diffraction peaks are measures of long-range structural order, while the inelastic scattering background can reveal transient phonon populations, as recently explored using UEDS^26–31,66–68^. Here, using first-order perturbation theory, the diffuse background intensity is given by^69^

1 $${I}_{1}({{{\bf{Q}}}})=\mathop{\sum}\limits_{\nu }\frac{\hslash }{2{\omega }_{{{{\bf{q}}}}\nu }}(2{n}_{{{{\bf{q}}}}\nu }+1)| {F}_{\nu }({{{\bf{Q}}}}){| }^{2},$$

where *n*_**q***ν*_, *ω*_**q***ν*_ and *F*_*ν*_(**Q**) are the population, frequency and structure factor of the phonon mode *ν* with the wave vector **q** = **Q** − **G**, in which **G** is the reciprocal lattice vector nearest to **Q**. The phonon structure factor is written as

2 $${F}_{\nu }({{{\bf{Q}}}})=\mathop{\sum}\limits_{\kappa }{f}_{\kappa }({{{\bf{Q}}}})\frac{{e}^{-{W}_{\kappa }({{{\bf{Q}}}})}}{\sqrt{{M}_{\kappa }}}[{{{\bf{Q}}}}\cdot {{{{\bf{e}}}}}_{\kappa ,\nu }({{{\bf{q}}}})],$$

where **e**_*κ*,*ν*_(**q**) is the phonon polarization vector and *M*_*κ*_ is the mass of the *κ*th atom. The exponent of the Debye–Waller factor is defined by

3 $${W}_{\kappa }({{{\bf{Q}}}})=\frac{\hslash }{4{M}_{\kappa }{N}_{p}}\mathop{\sum}\limits_{{{{\bf{q}}}}\nu }\frac{1}{{\omega }_{{{{\bf{q}}}}\nu }}(2{n}_{{{{\bf{q}}}}\nu }+1)| {{{\bf{Q}}}}\cdot {{{{\bf{e}}}}}_{\kappa ,\nu }({{{\bf{q}}}}){| }^{2}.$$

Here, the amplitudes *f*_*κ*_(**Q**) for the Ti and Se atoms were obtained numerically using the ELSEPA code^70^ for elastic scattering of electrons by atoms.

The relative contribution of each phonon mode to the diffuse background (cf. Fig. 2b) is estimated as follows:

4 $${w}_{{{{\bf{q}}}}\nu }=\frac{\frac{1}{{\omega }_{{{{\bf{q}}}}\nu }}(2{n}_{{{{\bf{q}}}}\nu }+1)| {F}_{\nu }({{{\bf{Q}}}}){| }^{2}}{{\sum }_{{{{{\bf{q}}}}}_{\perp }\nu }\frac{1}{{\omega }_{{{{\bf{q}}}}\nu }}(2{n}_{{{{\bf{q}}}}\nu }+1)| {F}_{\nu }({{{\bf{Q}}}}){| }^{2}}.$$

### Data analysis

#### Main lattice spots and diffuse background

The intensity of the main lattice peaks *I*_main_ is background corrected; that is, the raw peak intensity integrated within a circular area has to be reduced by the intensity average within a surrounding ring. After averaging the four main lattice peaks, the transient increase of the out-of-plane MSD $\langle {u}_{\perp }^{2}\rangle$ is calculated by

5 $$\Delta \langle {u}_{\perp }^{2}\rangle (\Delta t)=-\log \left(\frac{{I}_{{{{\rm{main}}}}}(\Delta t)}{{I}_{{{{\rm{main}}}},0}}\right)/{Q}^{2},$$

where *I*_main,0_ is the intensity at negative pump–probe delays and *Q* is the amplitude of the scattering vector. It is modelled by a stretched exponential function

6 $$\Delta \langle {u}_{\perp }^{2}\rangle (\Delta t)=a \Theta (\Delta t) [1-\exp (-{(\Delta t/{\tau }_{0})}^{\beta })],$$

determining also the temporal overlap Δ*t* = 0 between optical–pump and electron–probe pulses. Here, *a* is the total, laser-induced increase of the out-of-plane MSD, *Θ*(Δ*t*) is the Heaviside function, *τ*_0_ is the time constant and *β* ∈ (0, 1) is the stretching exponent. For a better comparability within the fluence-dependent measurements, we fit those datasets together, ensuring a common value for *β*.

Overall, the diffuse background stems from several contributions, namely (1) inelastic scattering with phonons (in other words, dynamical disorder), (2) the tail of the Bragg peaks (instrument response function) as well as (3) elastic scattering at static disorder (such as structural defects, adsorbates, grain boundaries and other point defects). While the phonon part increases after optical excitation, the Bragg part decreases. There are several equivalent ways to model the resulting transient evolution of such a multi-component diffuse background. We chose to describe absolute intensities, motivated by the fact that, in the description of the Debye–Waller dynamics of the Bragg peak, the determination of the MSD and phonon populations, ratios of intensities are the most relevant quantities. Moreover, this allows for a better representation of the observed dynamics across different rings, as shown in Fig. 3c, where we normalized the intensity to its value before photoexcitation. The opposing behaviour of different components in the background necessitates determining their amplitudes, including the third contribution which—together with the Bragg tail—is present before photoexcitation. We thus fit the intensity *I*_*q*_(Δ*t*) within a ring of momentum *q* by the following sum

7 $$\begin{array}{rcl}{I}_{q}(\Delta t)&=&{A}_{q} \Theta (\Delta t) [1-\exp (-\Delta t/{\tau }_{q})]+{B}_{q} {I}_{{{{\rm{main}}}}}(\Delta t)+{C}_{q},\end{array}$$

yielding parameters in the form of amplitudes *A*_*q*_, *B*_*q*_ and *C*_*q*_ as well as the phonon rise time *τ*_*q*_. The amplitudes resemble the strength of the three discussed intensity contributions.

#### Superstructure peaks

The structural order parameter *η* of the CDW is obtained from the similarly background-corrected intensity of three superstructure peaks *I*_2×2_ (indicated in the schematic diffraction pattern in Fig. 5b), also taking into account the Debye–Waller factor deduced from the intensity of the main lattice peaks *I*_main_

8 $$\frac{\eta (\Delta t)}{{\eta }_{0}}=\sqrt{\frac{{I}_{2\times 2}(\Delta t)}{{I}_{2\times 2,0}}/\frac{{I}_{{{{\rm{main}}}}}(\Delta t)}{{I}_{{{{\rm{main}}}},0}}},$$

whereby *η*_0_, *I*_2×2,0_ and *I*_main,0_ denote the respective values at negative pump–probe delays. Here, we can neglect *η*_0_ since the base temperature is far below *T*_c_ = 200 K and so *η*_0_ = *η*(30 K) ≈ 1. The temporal evolution of the order parameter *η*(Δ*t*) after the quench is modelled by the following exponential recovery towards a reduced, but thermalized value 1 − Δ*η*_therm_:

9 $$\begin{array}{rcl}\eta (\Delta t)&=&1-\Theta (\Delta t) \left[\Delta {\eta }_{{{{\rm{therm}}}}}\right.+\left.\Delta {\eta }_{{{{\rm{relax}}}}} \exp (-\Delta t/{\tau }_{{{{\rm{relax}}}}})\right],\end{array}$$

with the amplitude Δ*η*_relax_ and the time constant *τ*_relax_. A convolution of this function with the temporal resolution in the experiment is fitted to the data. The retrieved relaxation amplitude at the fluence threshold is identified with the excitonic contribution to the total lattice distortion, which is transiently completely suppressed.

### DFT calculation

Ab initio density functional calculations were performed using the QUANTUM ESPRESSO suite of codes^71,72^ including phonons from perturbation theory^73^ and their anharmonic properties^74^. The generalized gradient approximation with the Perdew–Burke–Ernzerhof exchange correlation functional^75^ and optimized norm-conserving pseudopotentials taken from the SG15 library^76,77^ were employed to calculate the ground state of the electronic system. The crystal structure was fully relaxed, and the resulting lattice parameters *a* = 3.49 Å and *c* = 5.97 Å are in a good agreement with the corresponding experimental values^78^ of 3.54 Å and 6.01 Å. A uniform grid of 16 × 16 × 6 and 4 × 4 × 2 points was used for sampling the Brillouin zone for electrons and phonons, respectively. For the Debye–Waller and structure factor calculation, we interpolated the phonon frequencies and polarization vectors on a finer grid of 100 × 100 × 50 points. The lifetimes of phonons, arising from anharmonic phonon–phonon interaction, were obtained using an integration grid of 220 × 220 × 100 points with a Gaussian broadening of 3 cm^−1^. The convergence has been tested using smaller smearing values and finer grids.

## Online content

Any methods, additional references, Nature Portfolio reporting summaries, source data, extended data, supplementary information, acknowledgements, peer review information; details of author contributions and competing interests; and statements of data and code availability are available at 10.1038/s41563-024-01880-6.

## Data Availability

The data shown in the manuscript are available via Edmond—the Open Research Data Repository of the Max Planck Society at 10.17617/3.JBZHN2 (ref. ^79^).
